# Transcriptomic analysis on pancreatic adenocarcinoma patients uncovers KRAS-mediated PPAR pathway alteration

**DOI:** 10.3389/fonc.2025.1613773

**Published:** 2025-08-11

**Authors:** Giuseppe Defazio, Federico Scolari, Sara Fancelli, Simone Polvani, Daniele Lavacchi, Lucia Picariello, Alessandro Tubita, Michaela Luconi, Lorenzo Antonuzzo, Andrea Galli, Serena Pillozzi

**Affiliations:** ^1^ Department of Experimental and Clinical Biomedical Sciences “Mario Serio”, University of Florence, Florence, Italy; ^2^ Clinical Oncology Unit, Careggi University Hospital, Florence, Italy; ^3^ Department of Experimental and Clinical Medicine, University of Florence, Florence, Italy

**Keywords:** pancreatic cancer, KRAS mutations, PPAR signaling pathway, transcriptomic analysis, lipid metabolism, molecular profiling

## Abstract

The incidence and mortality of pancreatic adenocarcinoma (PC) are expected to increase in the coming years, with survival rates remaining poor due to limited treatment options. KRAS mutations, present in over 70% of PC cases, drive aggressive tumor behavior through metabolic reprogramming and immune evasion; however, clinically effective inhibitors for the most common mutations are still lacking. In this study, we analyzed RNA sequencing data from TCGA datasets, comparing tumor versus normal pancreatic tissues and stratifying samples based on KRAS mutation status. Our findings reveal significant dysregulation of the peroxisome proliferator-activated receptor (PPAR) signaling pathway in PC, particularly in the context of KRAS mutations. These findings were validated through RT-qPCR in an independent cohort of primary samples. Key genes, including *CD36*, *FABP4*, *PLIN1*, *PLIN4*, *SCD5*, and *ACSLs*, were consistently downregulated in tumor tissues, with further reductions observed in KRAS-mutated samples. Overall, this study highlights the critical role of PPAR pathway disruption in KRAS-mutated PC, which should be further addressed to improve current treatment strategies.

## 1 Introduction

In the next 20 years the incidence of pancreatic adenocarcinoma (PC) is set to double especially in developing countries, and mortality estimates more than doubling (1). The range of available treatment options is still restricted to polychemotherapy, which is frequently poorly tolerated due to the rapid deterioration in patients’ clinical conditions. Consequently, the survival rate in advanced PC remains poor, with a median survival time of less than one year (1).

According to the International Cancer Genome Consortium (ICGC) data portal project, substitutions in Kirsten rat sarcoma virus gene (*KRAS*) occur in 78% of PC, and of them 70% are single-base missense substitutions on codon 12, with G12D (40%), G12V (31%), and G12R (19%) being the three most common mutations (2). Retrospective analyses have shown non-univocal prognoses among *KRAS* mutations although G12D and G12R seem to have the worst (3–5). Moreover, except for G12C hotspot mutations that account roughly 1% of cases (6), there are no clinically successful inhibitors for the most common mutations. Multi-selective RAS inhibitors (e.g. RAS[ON] inhibitors) are currently under investigation in clinical trials (NCT05379985, NCT04678648). Different combinations of drugs involved in downstream pathway inhibition, such as *SOS* and *SHP2* inhibitors, are able to reduce adaptive escape mechanisms via *MAPK* in *KRAS* mutant or amplified cancer cells in gastric cancer cell lines *in vitro* and *in vivo*. Moreover, KRAS mutations have been shown to promote immune escape in pancreatic cancer cells by suppressing both the number and activity of T cells, through specific immune-evading mechanisms associated with individual KRAS variants. Several experiences highlighted that, in addition to *KRAS* mutations, other factors, like changes in the tumor microenvironment (TME) supported by chronic inflammation, insulin resistance, a fatty diet, or factors associated with obesity, may increase *KRAS* activation and metabolic reprogramming (7). This reprogramming is fundamental in PC progression, involving several metabolic pathways, mainly glucose, glutamine, and fatty acid ones (8). At last, *KRAS* mutations also impact lipid metabolism. They can upregulate proteins such as FGL1, which alter lipid metabolism and enhance the proliferation of PC cells (9, 10). A high-fat diet has been shown to exacerbate the effects of *KRAS* mutations, further promoting the metabolic reprogramming of PC (11). *KRAS* mutations also affect other metabolic pathways, including the synthesis and utilization of acetyl coenzyme A and branched-chain amino acids. These alterations contribute to the aggressive nature of PC by supporting cellular proliferation and survival under nutrient-deprived conditions (8). Moreover, oncogenic *KRAS* reduces pancreatic *FGF21* expression, a metabolic regulator that prevents obesity, partially through downregulating peroxisome proliferator-activated receptor (*PPAR*)*G* (12).

Despite its rarity, *KRAS* wild-type (WT) PC represents a distinct molecular subtype with unique features. Retrospective analyses have shown no difference in Overall Survival (OS) between *KRAS*-WT and *KRAS* mutant PCs, regardless of the chemotherapy regimen used (4, 13). Recent evidence has demonstrated a distinct genomic profile in *KRAS*-WT PCs, identifying specific subgroups; these include forms with extrinsic *MAPK* pathway activation (e.g. *BRAF* mutation), those with microsatellite instability (MSI)/defective DNA mismatch repair (dMMR), and PCs with kinase fusion genes (14–16). The recent study by Singhi et al. showed that *MAPK* signaling is activated in approximately one-third of *KRAS*-WT PCs (17). In this group, *BRAF* mutations were the most common, however V600 mutations account for about 20% of the total limiting the possible use of target therapies to few cases. Additional mechanisms involved in *MAPK* activation have been identified, including gene mutations or amplifications in the *GNAS*, *EGFR*, *ERBB2*, *MET*, *ERBB3*, and *FGFR2* genes (18). MSI/dMMR PCs have a prevalence of 0.1 to 7% and exhibit a lower frequency of *KRAS* mutations than conventional PCs. MSI/dMMR PCs are more commonly observed in specific histotypes, including medullary carcinomas, mucinous/colloid variants, and IPMN-derived carcinomas (19). Additionally, approximately 8% of genetic alterations in all *KRAS*-WT PCs were identified as fusions of specific kinases, including those in *FGFR2*, *RAF*, *ALK*, *RET*, *MET*, *NTRK1*, and *FGFR3*. In patients with *KRAS*-WT PC and specific kinase fusions, targeted therapies such as afatinib for *NRG1* fusion, crizotinib for *MET* fusion, and erdafitinib for *FGFR2* fusion have been observed to elicit durable responses (15, 20).

In view of the considerable number of mechanisms involved in the development and progression of PC that are primarily driven by *KRAS*, we aimed to examine the transcriptomic distinctions between *KRAS*-mutated and *KRAS*-WT PCs using both a comprehensive transcriptomic approach on public datasets and quantitative real-time reverse transcription PCR (RT-qPCR) analysis on an independent cohort of primary PC.

## 2 Materials and methods

### 2.1 PC datasets

The RNA seqencing data used in the present manuscript were provided by The Cancer Genome Atlas (TGCA) as raw read counts obtained by the alignment of RNAseq reads against the Human reference genome (GRCh38) to obtain gene expression profiles. The data provider aligned RNAseq reads against reference using STAR (21) to infer raw read counts for mRNAs. To facilitate harmonization across samples, all RNA-Seq reads were treated as unstranded during analyses (22). The sample data and metadata were retrieved by using the Application Programming Interface (API) of Genomic Data Commons Data Portal (GDC, accessed on 17/11/2022) wrapped in a Python 3 in-house developed script (https://github.com/gdefazio/TCGA_pancreas). This allowed the selection of freely available datasets with “Pancreas” as primary site and labeled as “Primary Tumor” or “Solid Tissue Normal’’ (i.e. the tumor-adjacent normal tissue). Gene expression profiles for 367 tumor vs 72 adjacent normal tissue samples were locally collected. Furthermore, in order to investigate the difference in transcriptome profiles among *KRAS* mutated and *KRAS*-WT tumors the Whole Exome Sequencing (WES) data from GDC API were retrieved.

### 2.2 Unpaired, Paired and *KRAS*-related group analysis

Expression profile analyses were performed comparing either all the 367 tumor samples with all the 72 adjacent normal tissue samples (unpaired analysis) or in a subset of 42 patients comparing each tumor with its adjacent normal tissue samples (paired analysis). In the paired analysis, *KRAS* mutated versus WT tumor samples were also compared.

### 2.3 Identification of differentially expressed genes

A noise reduction strategy was implemented for gene expression data by eliminating genes with a read count ≤10 in more than half of the total samples.

The differential expression analysis was performed by using DESeq2 (v 1.34.0) R package (23). DESeq2 allows to indicate terms of comparison in the experimental design formula. In order to take into the account batch effect of data from different TCGA centres also this label was included in the experimental design formula as suggested in (24). For pairwise comparison only, patients’ case identifier was included in the experimental design formula and batch effect was not with the aim to avoid the “Model matrix not full rank” error (i.e. linear combination of terms) explained in (24).

P-values were adjusted with the Bonferroni method to avoid false-positive results and the 50 most up and down regulated genes with adjusted p-values ≤ 0.05 were taken as differentially expressed.

For DEGs heatmap graphical representation, before the z-score normalization, the batch effect was reduced by using the removeBatchEffect function in the limma (v 3.50.3) R package (25) on gene counts. This was performed only for the analyses in which batch effect was included in the experimental design formula.

### 2.4 KEGG enrichment analysis

Kyoto Encyclopedia of Genes and Genomes (KEGG) pathway analysis was performed on the lists of up- and down-regulated DEGs using ClusterProfiler (v. 4.2.2) R package (26). Benjamini-Hochberg adjusted p-value was computed and only significantly enriched pathways with more than 10 genes were selected.

### 2.5 Patients and biopsy processing

Surgical specimens were collected from 18 patients with pathologically confirmed PC who underwent surgical resection for operable disease and referred to the Clinical Oncology Unit, Careggi University Hospital, Florence (Italy). The recruitment period was from 23.03.2023 to 09.01.2024. All participants gave written informed consent before enrollment. Patients were excluded if they had metastatic or locally advanced inoperable disease or if they were under 18 years old.

### 2.6 Cell lines, drugs and viability assays

*KRAS*-WT, *KRAS*-p.G12C and *KRAS*-p.G12D PC cell lines (BxPC3, MiaPaca-2 and Panc-1 respectively) were obtained from the American Tissue Type Collection and cultured as previously reported (27). MiaPaca-2 and Panc-1 were maintained in Dulbecco’s Modified Eagle’s Medium (DMEM) with 10% foetal bovine serum (FBS), 2 mM glutamine, 50 U/mL penicillin and 50 mg/mL streptomycin (Euroclone, Milan, Italy) at 37°C and 5% CO2. The presence of mycoplasma was periodically tested by PCR. Cell viability was measured using Prestoblue™ Cell Viability reagent (Invitrogen, Waltham, MA, USA) according to the manufacturer’s protocol. The optical density (OD) was measured using a 560nm excitation filter and 590nm emission filter using the BioTek Synergy™ H1 hybrid multi-mode microplate reader (Agilent, CA, USA). The PPARG inhibitor used in this work was GW9662. The *KRAS* inhibitor used was Sotorasib. Cells were treated with these agents at the corresponding IC50 concentration (13nM for Sotorasib, 9*µ*M for GW9662, both determined at 72h) alone or in combination for 48 hours. Sotorasib and GW9662 were purchased from MedChemExpress (Monmouth Junction, NJ, USA).

### 2.7 RNA extraction and RT-qPCR

A total of 18 tumor samples of enrolled patients and 13 pancreas tissue samples from healthy donors were used for the analysis of a panel of genes, namely *CD36, FABP4*, *PPARA, PPARD*, *PPARG, PLIN1*, *PLIN4*, *SCD5 and ACSL4*. Total RNA was extracted from FF cryosections using the Qiagen RNeasy FFPE extraction.

BxPC3, MiaPaca-2 and Panc-1 cell lines were also used for the analysis of the above genes. Total RNA was extracted from cells using TRIzol reagent (Life Technologies, MI, Italy).

The RNA quantity and purity were evaluated using a Nanodrop spectrophotometer. All mRNAs were retro-transcribed using the Reverse Transcriptase kit 2 (EXPERTEAM, VE, ITALY); RT-qPCR analysis was performed on ABI7000 (Applied Biosystem, Foster City, CA, USA) using QuantiNova SYBR Green PCR Kit (Qiagen, MI, Italy). The primers used were:

*GAPDH* (QuantiTect Primer Assay QT00079247, Qiagen); *YWHAZ* (QuantiTect Primer Assay QT00087962, Qiagen); *CD36* (QuantiTect Primer Assay QT01974008, Qiagen); *FABP4* forward (5’-ACGAGAGGATGATAAACTGGTGG-3’) reverse (5’- GCGAACTTCAGTCCAGGTCAAC-3’); *PPARA* forward (5’-TCGGCGAGGATAGTTCTGGAAG-3’) reverse (5’-GACCACAGGATAAGTCACCGAG.-3’); *PPARD* forward 5’-GGCTTCCACTACGGTGTTCATG-3’) reverse (5’-CTGGCACTTGTTGCGGTTCTTC-3’); *PPARG* (QuantiTect Primer Assay QT00029941, Qiagen); *PLIN1* forward (5’-GCGGAATTTGCTGCCAACACTC-3’) reverse (5’-AGACTTCTGGGCTTGCTGGTGT-3’); *PLIN4* forward (5’-GATGGCAGAGAACGGTGTGAAG-3’) reverse (5’-CAGGCATAGGTATTGGCAACTGC-3’); *SCD5* forward (5’-GAGGAATGTCGTCCTGATGAGC-3’) reverse (5’- GCCAGGAGGAAGCAGAAGTAGG-3’); *ACSL4* forward (5’- GCTATCTCCTCAGACACACCGA -3’) reverse (5’-AGGTGCTCCAACTCTGCCAGTA-3’). Each primer was used at 200nM concentration (400nM finale for pairs). Cycle conditions were as follows: initial activation/denaturation 95°C 1’; 40 cycles of: 95°C 15”, 60°C for 1’; standard melting cycle for Applied ABI 7000.

The relative quantification was performed using *GAPDH* and *YWHAZ* as housekeeping genes. ΔCt values in tumor and healthy tissue samples were compared with a Wilcoxon rank-sum test.

### 2.8 Ethics and regulatory considerations

The present study was approved by the Regional Ethics Committee for Clinical Trials of the Tuscany Region (Firenze, Italy; no. 23753_BIO). All informed consent documents were in compliance with the International Conference on Harmonization (ICH) guideline on good clinical practice (GCP). The study protocol was performed in accordance with the principles of the Declaration of Helsinki and in compliance with GCP and the applicable laws and regulations. Each patient was identified by a code instead of the patient’s name in order to protect the patient’s identity when reporting study-related data.

## 3 Results

### 3.1 Tumor versus normal pancreatic tissue unpaired analysis

**Figure 1:**
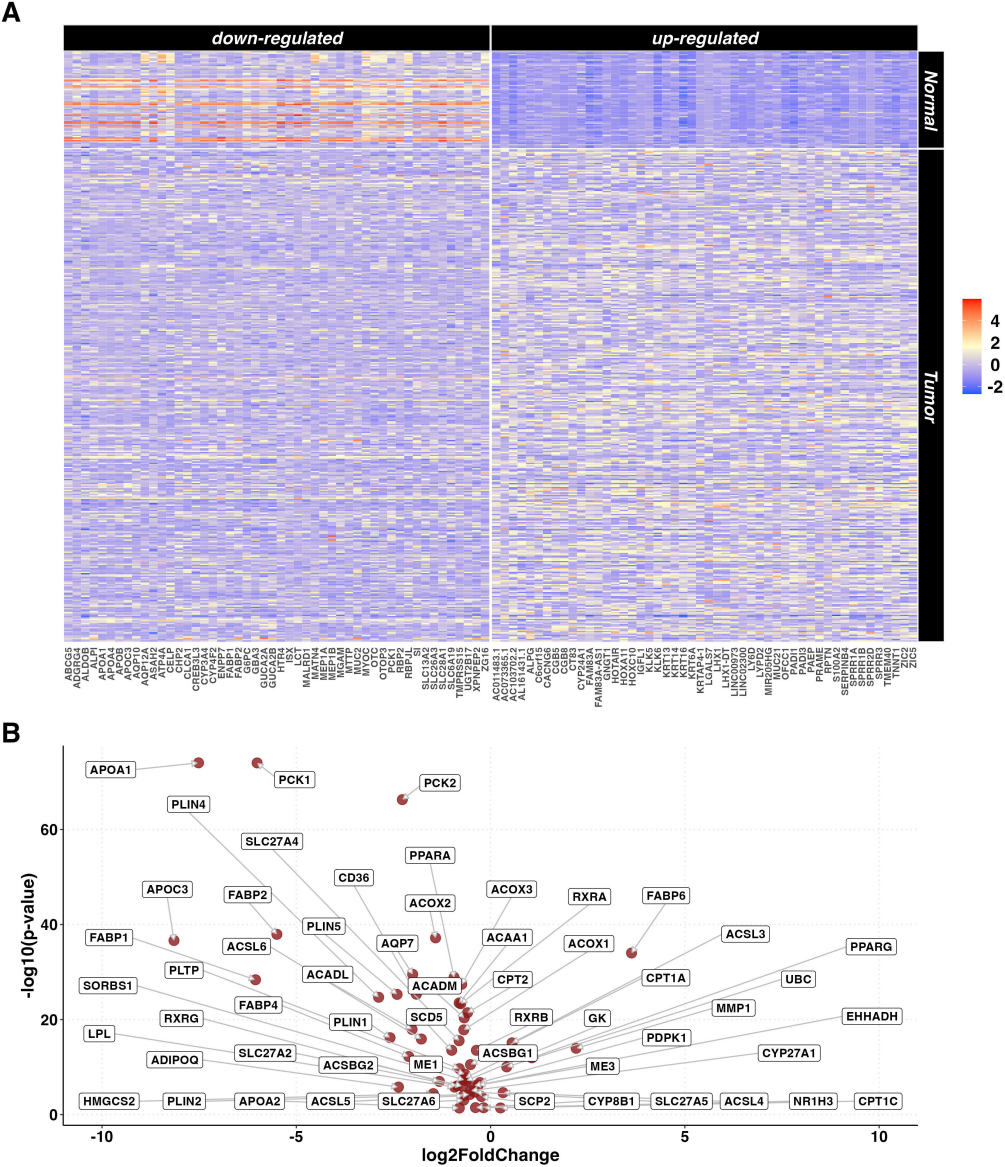
DEGs in tumor vs normal tissue unpaired analysis. **(A)** Heatmaps representing z-score transformed counts for the most 50 up- (right sided) and down- (left sided) regulated genes in the PC (n=367) vs normal tissue (n=72) unpaired comparison. **(B)** Volcano plot reporting only the differentially expressed genes related to the *PPAR* Signaling Pathway in the PC (n=367) vs normal tissue (n=72) unpaired comparison.

Gene expression data of 367 primary tumors of PC and 72 normal tissue samples were retrieved from 4 different TCGA projects (**Supplementary Table S1**). A total of 21,412 DEGs including 6,727 up- and 14,685 down-expressed were identified by tumor versus normal tissue comparison. Of these, 55% were protein coding, 26% were lncRNA and 9% were processed pseudogenes. KEGG pathways over-representation analysis (ORA) was performed both on the up- and down-regulated genes, resulting in60 and 66 enriched pathways, respectively (**Supplementary Table S2**). The 50 most up- and down-regulated genes are reported in **Figure 1A**. One of the most significantly over-represented pathways in the down-regulated list was *PPAR* signaling pathway (p.adjusted < 0.001). **Figure 1B** shows a Volcano plot indicating the specific DEGs related to the *PPAR* signaling pathway in the tumor vs normal samples.

### 3.2 Tumor versus normal pancreatic tissues paired analysis

**Figure 2:**
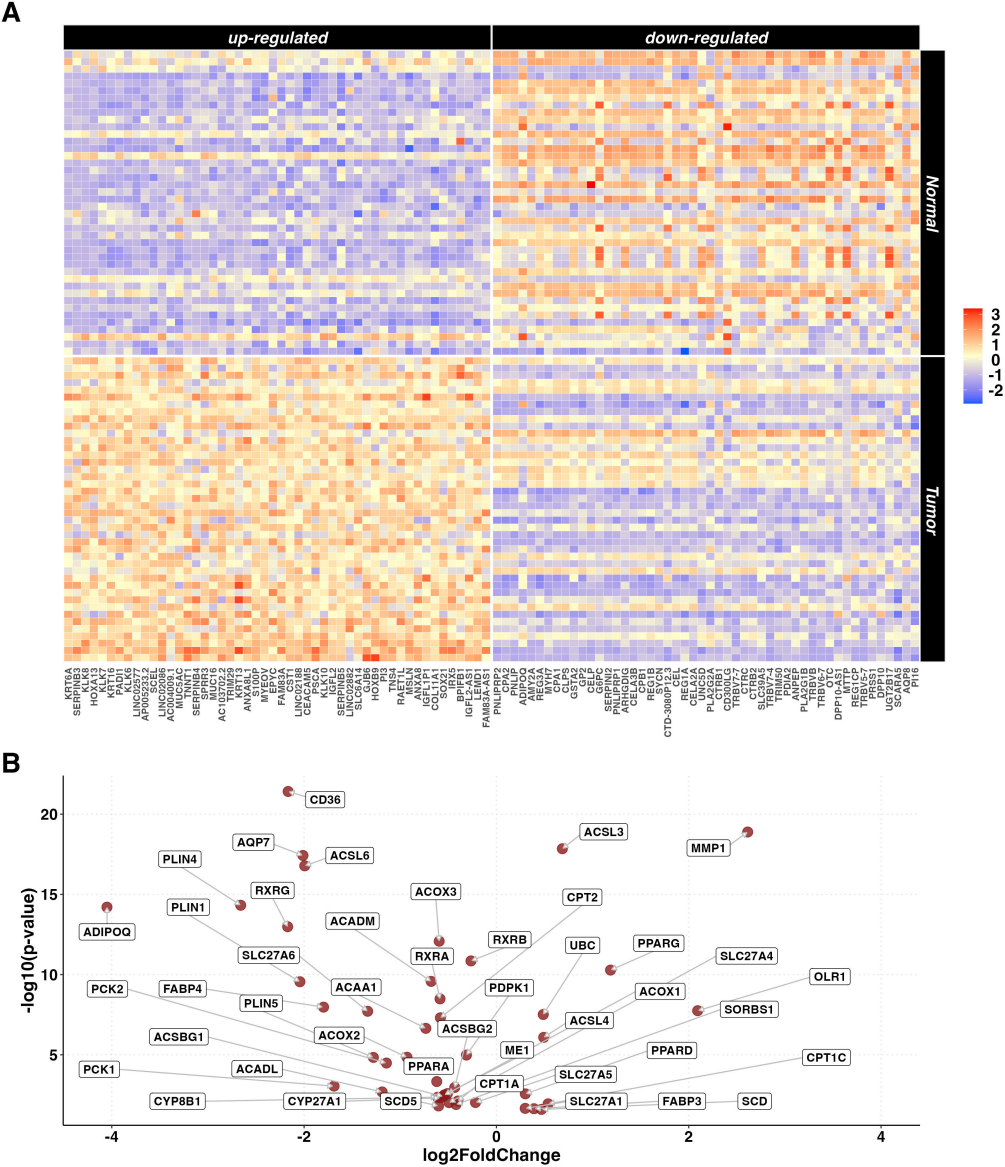
DEGs in paired tumor vs normal tissue analysis. **(A)** Heatmaps representing z-score transformed counts for the most 50 up- (left sided) and down- (right sided) regulated genes in the PC (n=42) vs normal (n=42) paired comparison. **(B)** Volcano plot reporting only the differentially expressed genes related to the *PPAR* Signaling Pathway in the paired PC (n=42) vs normal (n=42) comparison.

From the unpaired set, gene expression data of 84 samples (42 tumor and 42 adjacent normal tissue samples) belonging to 42 PC patients were selected. The paired comparison between tumor and adjacent normal tissue samples identified a statistically significant difference in the expression of 15,660 DEGs (6,608 up- and 9,052 down-regulated). Out of these, 63% were protein coding, 22% were lncRNA and 8% were processed pseudogenes. A heatmap representing the 50 most up- and down-regulated genes is reported in **Figure 2A**. KEGG pathway ORA revealed 64 enriched pathways for the upregulated genes and 35 for the downregulated genes (**Supplementary Table S3**), notably including *PPAR* signaling pathway (p=0.007). A Volcano plot showing the *PPAR*-related DEGs differentially expressed in the paired analysis is reported in **Figure 2B**.

To further investigate the role of the *PPAR* pathway in PC, differences in the expression of the individual genes related to this pathway were evaluated. Results showed that some of the most relevant pathway’s regulators and effectors (*CD36*, *FABP4*, *PLIN1*, *PLIN4*, *SCD5 and ACSL6*) showed significantly lower expression in tumor tissue samples (p.adjusted < 0.01, data not shown). Conversely, *PPARD and PPARG* showed significantly higher expression in tumor tissue samples, however, only *PPARG* exceeded the threshold of LogFC>1.

### 3.3 RT-qPCR validation in an independent PC cohort

**Figure 3:**
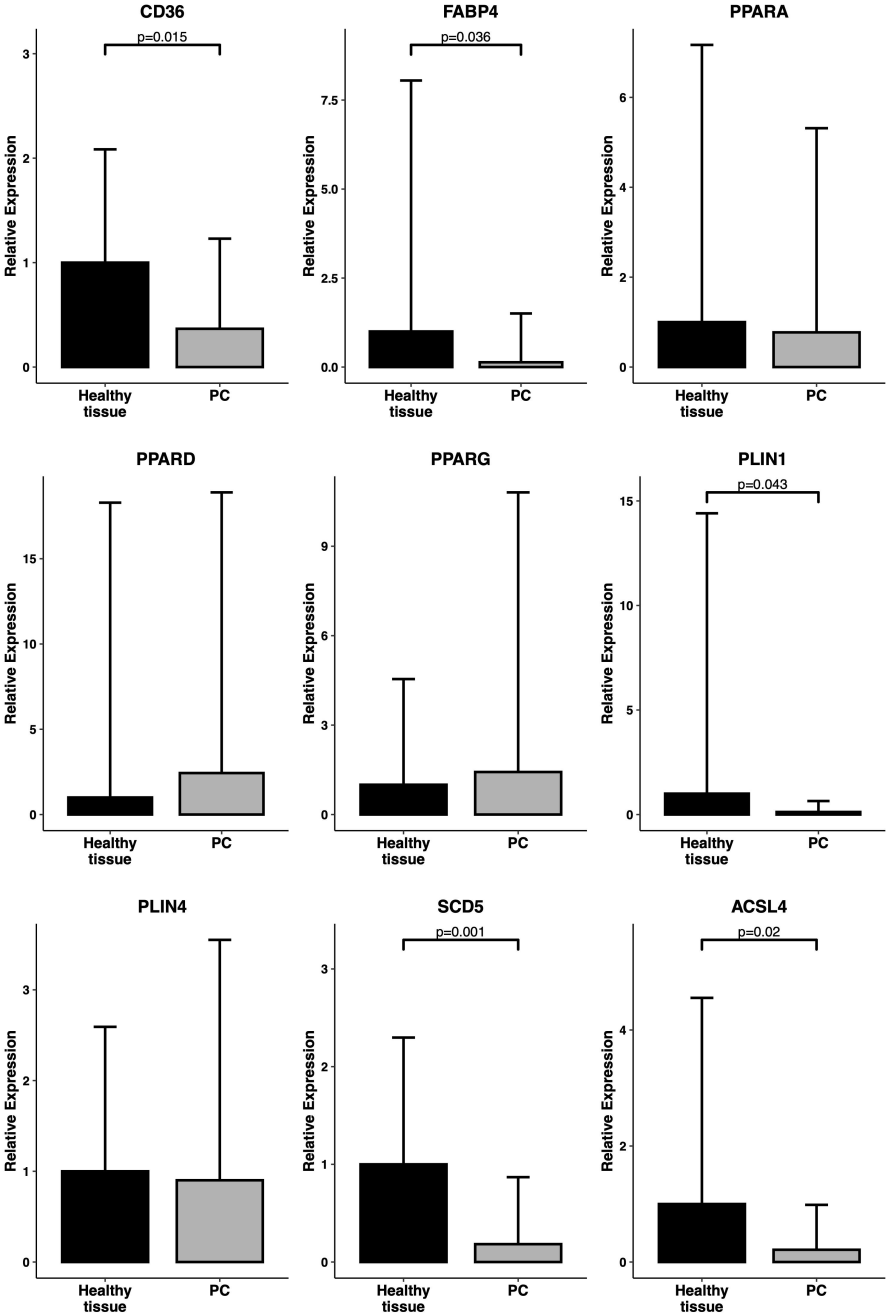
Expression levels of PPAR-related genes in primary samples. RT-qPCR analysis of a panel of *PPAR*-related genes (*CD36*, *FABP4*, *PPARA*, *PPARD*, *PPARG*, *PLIN1*, *PLIN4*, *SCD5, ACSL4*) in an independent cohort of 19 PC samples vs 13 healthy tissue samples. Relative expression is reported as 2^(-ddCT). P-values have been computed by comparison with a Welch’s t-test.

**Figure 4:**
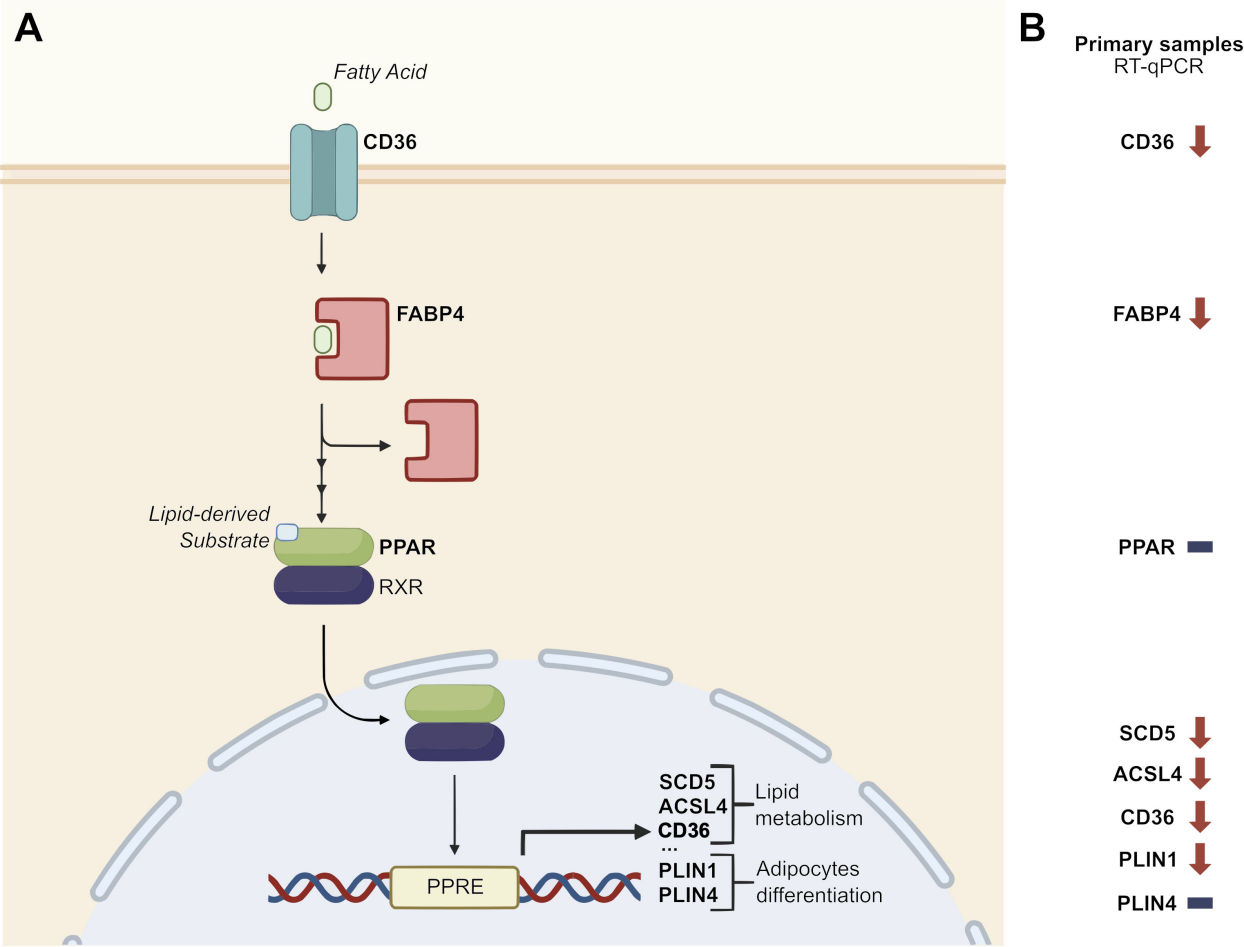
Disruption of PPAR signaling pathway. **(A)** Schematic representation of the role of a panel of *PPAR*-related genes in the *PPAR* signaling pathway (adapted from KEGG pathway hsa03320 – “*PPAR* signaling pathway – Homo sapiens”). **(B)** Focus on the relative expression of *PPAR*-related genes in an independent cohort of 19 PC samples vs 13 healthy pancreatic tissue samples. Downward arrows represent downregulation in tumor samples, horizontal lines represent no difference in tumor vs healthy tissue samples. PPRE: *PPAR* Response Element.

The differential expression signature identified by the bioinformatic analysis was validated by RT-qPCR analysis in an independent cohort of pancreatic tissue samples (19 primary tumors and 13 normal pancreatic tissue samples) obtained from 32 patients enrolled and operated at Careggi University Hospital. The comparative analysis was focused on the expression of a panel of genes related to the *PPAR* pathway, lipid metabolism and adipocyte differentiation, namely *CD36, FABP4, PPARD*, *PLIN1*, *SCD5* and *ACSL4*. Most of the genes showed expression patterns similar to those observed in the TCGA cohort analysis. Specifically, *CD36, FABP4*, *PLIN1*, *SCD5* and *ACSL4* were significantly downregulated in tumor samples (p < 0.05). Results are reported in **Figure 3**. A schematic representation of the *PPAR* pathway, with a particular focus on the genes considered in this analysis, is presented in **Figure 4**.

### 3.4 *KRAS* mutated versus WT PC analysis

**Figure 5:**
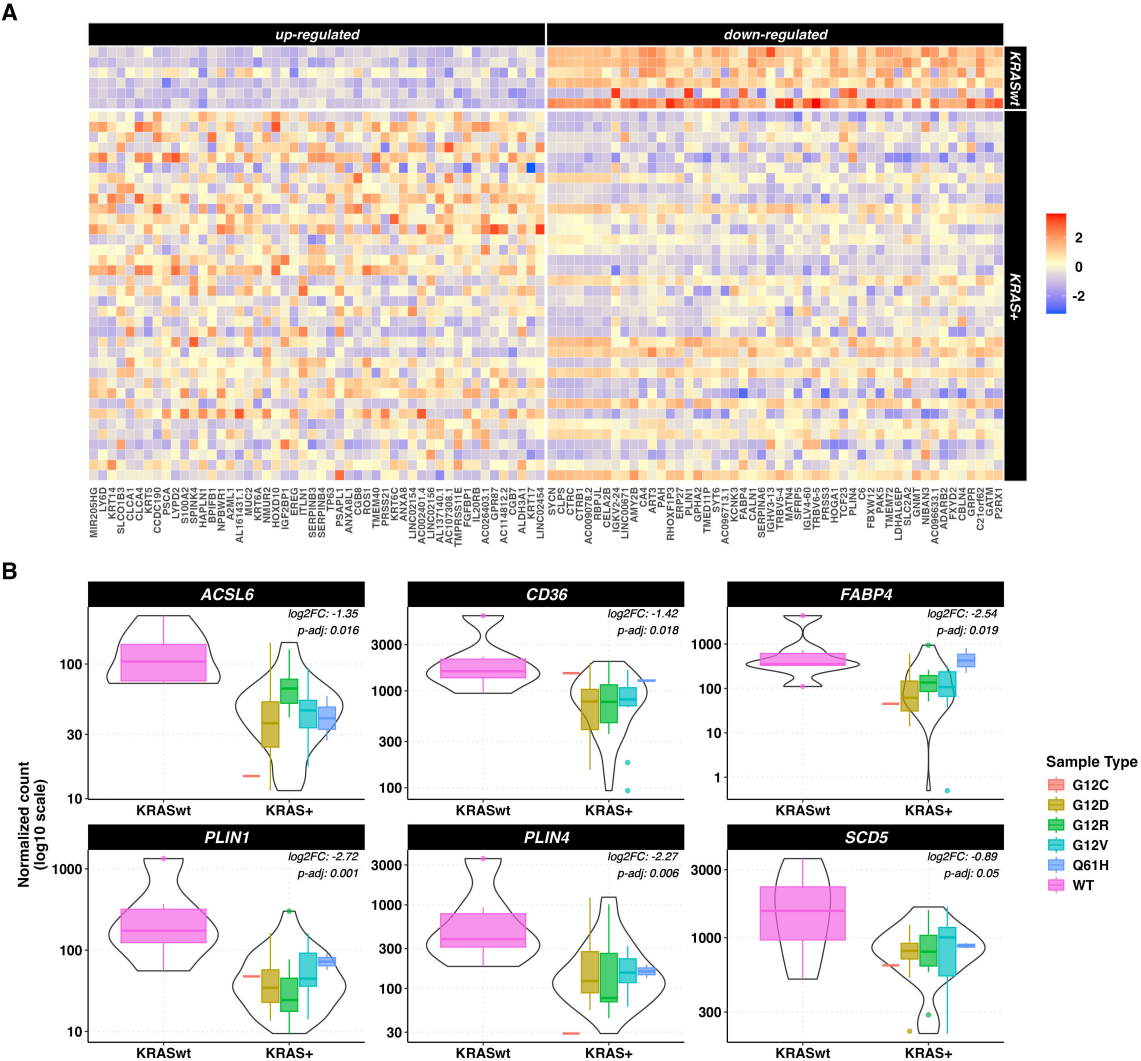
DEGs in tumor vs normal tissue unpaired analysis. **(A)** heatmaps representing z-score transformed counts for the most 50 up- (left sided) and down- (right sided) regulated genes between the KRAS-mutated (n=36) vs KRAS-WT (n=6) PC samples. **(B)** Expression levels of ACSL6, CD36, FABP4, PLIN1, PLIN4 and SCD5 in KRAS-mutated vs KRAS-WT pancreatic cancer samples measured as log10-scaled normalized counts. The KRAS mutated group is stratified according to the 5 different KRAS mutations. The p-values and log 2-Fold Changes (L2FC) refer to the KRAS mutated vs WT comparison.

Since *KRAS* mutation is considered a main oncogenic driver in the vast majority of PCs, we evaluated if the deregulation of the *PPAR* pathway could be associated with a specific *KRAS* mutation profile: Therefore, gene expression data of 6 *KRAS*-WT versus 36 *KRAS*-mutated tumor samples from the TCGA dataset were compared. The distribution of the hotspot mutations in the dataset was: n=16 p.G12D, n=10 p.G12V, n=7 p.G12R, n=2 p.Q61H and n=1 p.G12C. The number of DEGs between *KRAS*-mutated and *KRAS*-WT samples was 808: 388 genes were up- and 420 were down-regulated in the *KRAS*-mutated samples. Of these genes, 78% were protein coding, 13% were lncRNA and 3% were processed pseudogenes. Heatmaps showing the 50 most up- and down-regulated genes in *KRAS*-mutated samples are depicted in **Figure 5A**. KEGG pathway ORA showed one over-expressed pathway for the up-regulated genes and 5 pathways for the down-regulated genes (**Supplementary Table S4**). The *PPAR* signaling pathway was significantly over-represented in the down-regulated genes list (p=0.046).

Focusing on the *PPAR* signaling pathway related genes, 6 under-expressed genes were identified in *KRAS*-mutated versus *KRAS*-WT samples, namely *ACSL6, CD36, FABP4, PLIN1, PLIN4* and *SCD5* (p < 0.5). Results are shown in **Figure 5B**.

### 3.5 *PPAR* signature in PC cell line models

**Figure 6:**
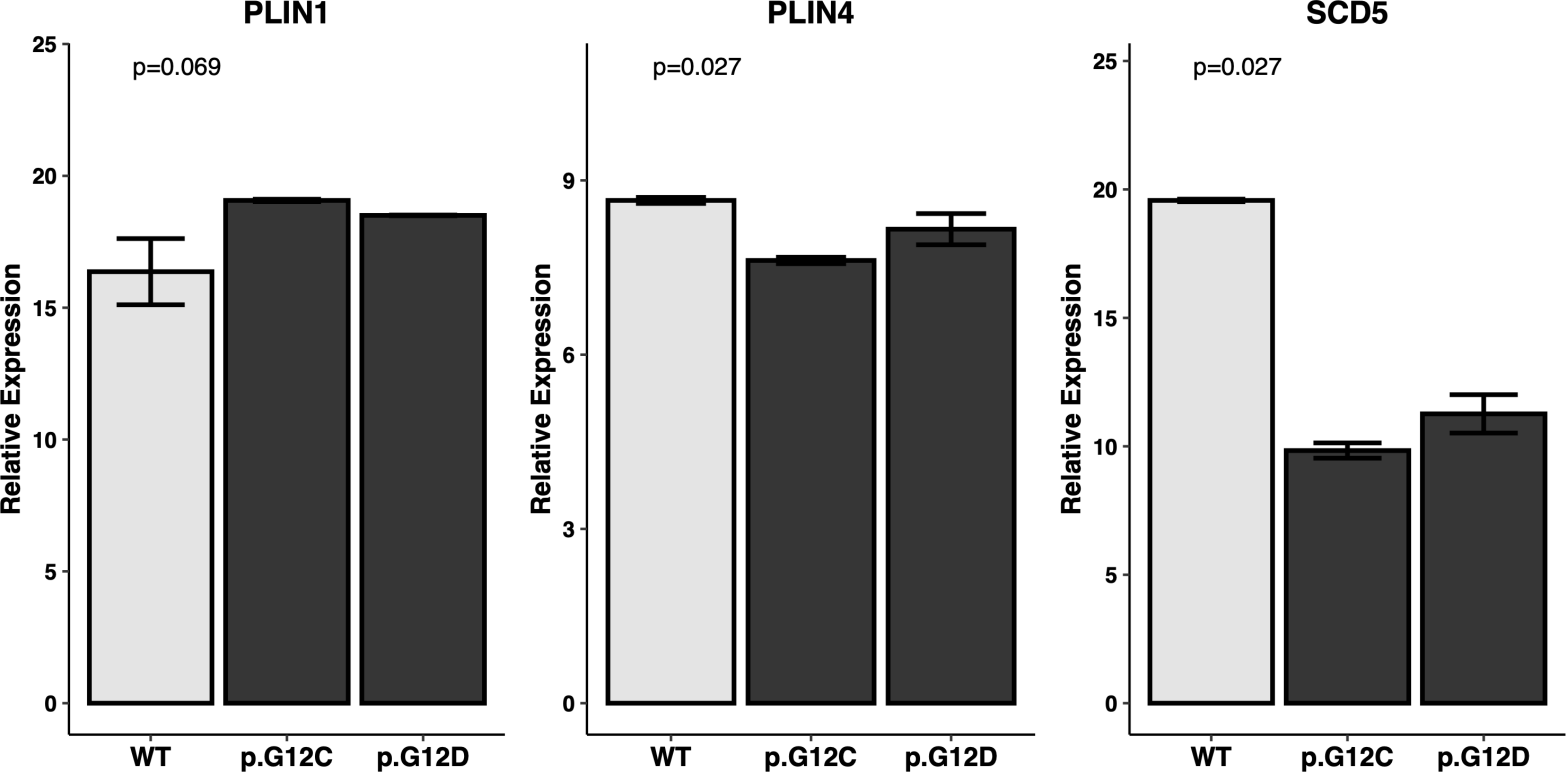
Expression levels of *PPAR*-related genes in PC cell lines. Expression of the PPAR-related genes of interest (*PLIN1*, *PLIN4*, *SCD5*) was assessed by RT-qPCR in a set of *KRAS* mutated and *KRAS* WT PC cell lines. Relative expression is reported as dCT against control genes. Statistical significance expressed by P value has been tested with Kruskall-Wallis’s test.

In order to confirm the results obtained from the *KRAS*-WT vs mutant analysis of PC, RT-qPCR analysis was performed on *KRAS*-WT, *KRAS*-p.G12C and *KRAS*-p.G12D PC cell lines. The influence of *KRAS* mutations on lipid metabolism and adipocyte differentiation was evaluated by analyzing the expression of *PPAR* pathway downstream effectors *PLIN1*, *PLIN4* and *SCL5*. A statistically significant downregulation of *PLIN4* and *SCD5* was evident in *KRAS*-mutated vs WT cell lines (p=0.027), while *PLIN1* showed no differences among all cell lines (**Figure 6**).

### 3.6 *PPAR* inhibitor in combination with *KRAS* p.G12C inhibitor reduces PC cell viability *in vitro*

To investigate the potential interaction between *KRAS* and *PPAR* signaling in PC, we evaluated the effect on cell viability in *KRAS* p.G12C and *KRAS* p.G12D mutated PC cell lines treated *in vitro* with the *KRAS* inhibitor Sotorasib alone or combined with the PPAR inhibitor GW9662. The results reported in **Supplementary Figure S1** show that cell viability was significantly reduced in the *KRAS* p.G12C mutated PC cell line after 48 hours of Sotorasib treatment (*p=0.026*) and to a greater extent when Sotorasib was combined with GW9662 (p=0.020). A similar inhibitor effect was observed in the *KRAS* p.G12D mutated PC cell line only after the combined treatment with Sotorasib and GW9662 (p=0.01).

## 4 Discussion

PC is considered one of the deadliest malignancies worldwide, with limited therapeutic options and a poor prognosis. The addition of novel agents, including immune-checkpoint inhibitors or stroma-targeting drugs, to standard chemotherapy provided disappointing results. Understanding the PC molecular mechanisms and pathogenesis is critical for developing new and more effective treatments.

In our study, over 120 KEGG pathways were found to be differentially expressed in tumor vs healthy tissue samples from a TCGA PC cohort. The subsequent analysis focused on the *PPAR* signaling pathway, which was significantly deregulated in both unpaired and paired analysis of the TCGA dataset. These findings have also been validated with RT-qPCR analysis in an independent cohort of primary PC samples, further suggesting the disruption of this signaling pathway in PC. Specifically, we observed a significant down-regulation of some of the most relevant genes in the *PPAR* pathway (*CD36, FABP4*, *PLIN1*, *PLIN4*, *SCD5* and *ACSL4*) in tumor tissue samples. A significant down-regulation of *CD36*, *FABP4*, *PLIN1*, *SCD5* and *ACSL4* in tumor samples has also been validated by RT-qPCR. These genes are involved in several mechanisms such as fatty acid transport, fatty acid metabolism and lipid droplet formation. Focusing on specific *PPAR* pathway receptors, we observed *PPARG* and *PPARD* upregulation in PC tumor tissue samples in the TCGA cohort. *PPAR* signaling dysregulation has been previously reported in various cancer types, including PC (28, 29). Several studies have provided insights into the role of specific *PPAR* genes in the context of metabolic reprogramming and tumor progression, but a consensus is still not defined. *PPAR* signaling pathway over-expression has been recently observed in metastatic vs primary PC samples of three different public datasets (30). Additionally, *PPARD* activation driven by metabolic stress and signals from tumor-associated macrophages (TAMs) has been shown to increase epithelial-mesenchymal transition (EMT) and enhance cancer cell invasiveness in *in vitro* and *in vivo* models (31); furthermore, *PPARD* activation by *GOT2* regulation in *in vitro* models has been linked to tumor progression and immune suppression (10). Conversely, *PPARD* activation has been correlated with the reduction of cell invasion and metastasis related genes in PC cell lines (32). Our findings are in line with a tumor-related role of *PPAR* genes, but the overall downregulation of the PPAR downstream pathways in tumor samples may reflect a more intricate signaling mechanism. Our analyses were conducted on bulk tumor RNA, therefore the observed alterations may represent a composite effect across multiple cell types, including cancer cells and surrounding stromal or immune cells.

The dichotomy between PPAR receptor over-expression and the downregulation of downstream signaling in PC cells may be attributed to an altered availability of PPAR endogenous ligands. This alteration could result from the downregulation of FABP4 and CD36, both of which play critical roles in lipid uptake in PC cells.

The GTPase *KRAS* is activated in over 80% of PC and is a driver of tumorigenesis and metabolic reprogramming (33). *KRAS* mutations are known to drive PC tumorigenesis through various signaling pathways (8, 14). Recent findings obtained both *in vitro* and *in vivo* show that *PPARD* has a pivotal role in promoting the tumorigenesis of *KRAS*-mutated pancreatic lesions by increasing the recruitment of pancreatic macrophages and myeloid-derived suppressor cells (MDSCs), thus promoting an immunosuppressive TME (34). Conversely, the activation of *PPARD* leads to the development of a tumor suppressive TME by inhibiting Th2/M2 differentiation (35). Furthermore, KRAS mutations have been associated with the downregulation of *PPARA* and *PPARG* (12), reinforcing the interaction between these pathways.

Our study revealed a significant correlation between *KRAS* mutations and the downregulation of the *PPAR* signaling pathway. Specifically, a panel of *PPAR*-related genes (*CD36*, *FABP4*, *PLIN1*, *PLIN4*, *SCD5*, and *ACSL4*) were significantly under-expressed in *KRAS*-mutated samples compared to WT samples. In our *in vitro* experiments, we further validated the influence of *KRAS* mutations on *PPAR* signaling. RT-qPCR experiments conducted on *KRAS* WT, *KRAS* p.G12C, and *KRAS* p.G12D cell lines confirmed a significant downregulation of *PLIN4* and *SCD5* in *KRAS*-mutated cell lines.

*KRAS* has been thought to promote a shift to aerobic glycolysis and anabolic glucose metabolism (36). However, our understanding of *KRAS*-driven metabolic reprogramming has evolved to include alterations in scavenging pathways, amino acid metabolism, and lipid metabolism (37). Our findings align with previous research indicating that *KRAS* mutations modulate metabolic pathways, including lipid metabolism, to support the energetic and biosynthetic demands of rapidly proliferating cancer cells (38).

The deregulation of the *PPAR* pathway in the context of *KRAS* mutations presents potential therapeutic opportunities. A recent study explored the Hippo-*FAM60A*-*PPAR* axis as a key regulator of ferroptosis and a therapeutic target in *KRAS*-mutated *PPAR* cell lines (39). *PPAR* agonists such as thiazolidinediones (TZDs) have been explored for their anti-tumor effects in various cancer types (40, 41); however, the clinical use for PC treatment did not yield successful results in clinical trials (42), maybe due to drug regimen which has been optimized for type-2 diabetes indication or to alternative nongenomic mechanisms described for this multifunctional receptor (43).

In conclusion, our study provides evidence for the significant deregulation of the *PPAR* signaling pathway in PC, particularly in the context of *KRAS* mutations, in both a public cohort and an independent cohort of primary tumor samples. The consistent down-regulation of key *PPAR*-related genes involved in lipid metabolism underscores potential metabolic vulnerabilities in *KRAS*-mutated PC, which could be exploited to develop more effective treatment strategies.

## Data Availability

The original contributions presented in the study are included in the article/**Supplementary Material**. Further inquiries can be directed to the corresponding author.
