# Supplementary material for: Transcriptomic analysis on pancreatic adenocarcinoma patients uncovers KRAS-mediated PPAR pathway alteration
Source: Front Oncol. 2025 Aug 11;15:1613773. doi: 10.3389/fonc.2025.1613773 (PMC12375456; doi:10.3389/fonc.2025.1613773)
Supplement: Supplementary file 1 [file DataSheet1.pdf]

Supplementary Figures

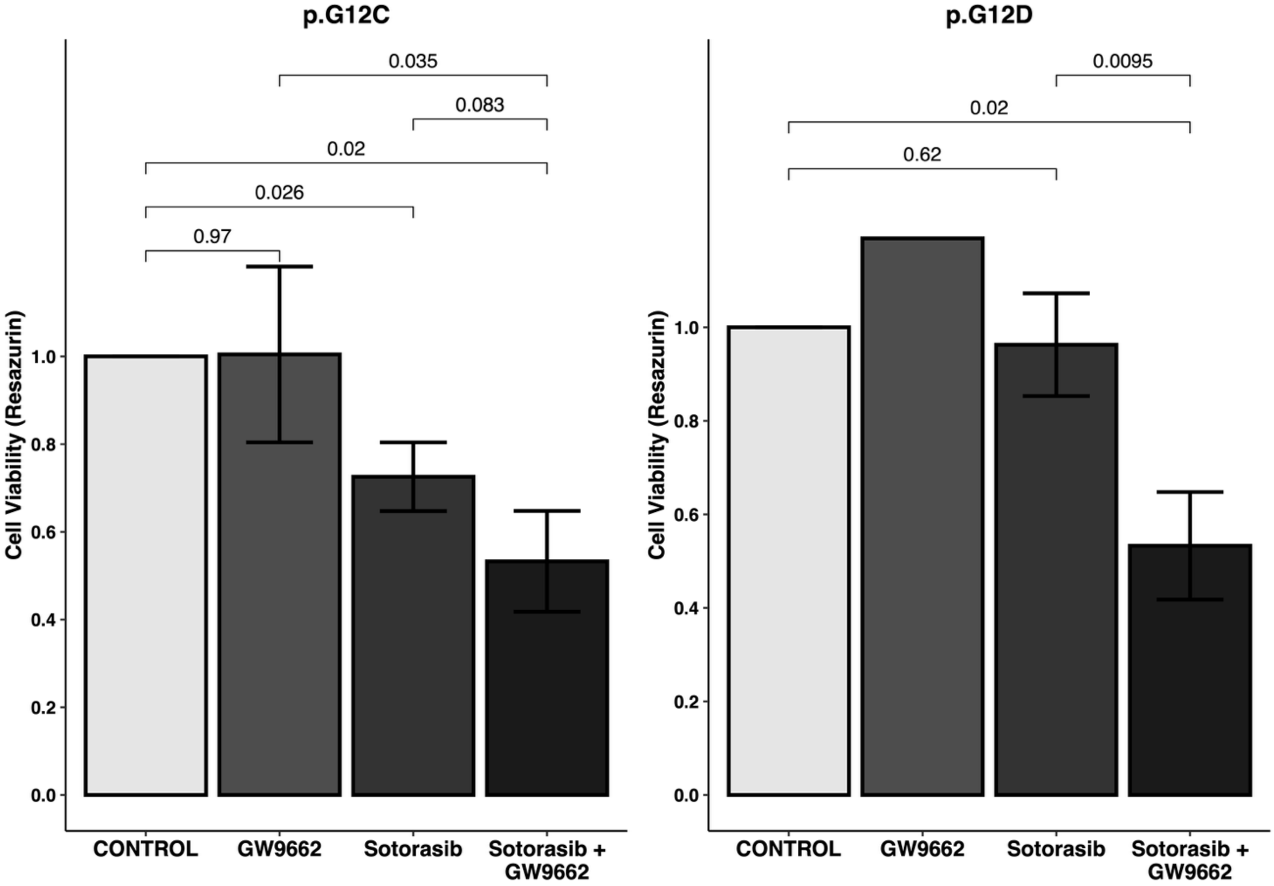

Figure S1. Cell viability in PC cell lines treated with KRAS and PPAR inhibitors. The combination of Sotorasib and GW9662 induces growth inhibition in KRAS p.G12C and KRAS p.G12D mutated PC cells. KRAS p.G12C mutated PC cells show sensitivity to Sotorasib and to the combination of Sotorasib and GW9662, while KRAS p.G12D mutated PC cells show sensitivity only to the combination of Sotorasib and GW9662. Statistical significance expressed by P-value have been computed with a Welch's t-test.
